# In human non-REM sleep, more slow-wave activity leads to less blood flow in the prefrontal cortex

**DOI:** 10.1038/s41598-017-12890-7

**Published:** 2017-11-03

**Authors:** Laura Tüshaus, Ximena Omlin, Ruth O’Gorman Tuura, Andrea Federspiel, Roger Luechinger, Philipp Staempfli, Thomas Koenig, Peter Achermann

**Affiliations:** 1 Chronobiology and Sleep Research, Institute of Pharmacology and Toxicology, University of Zurich, 8057 Zurich, Switzerland; 2 Neuroscience Center Zurich, University of Zurich and ETH Zurich, 8057 Zurich, Switzerland; 3 Zurich Center for Interdisciplinary Sleep Research, University of Zurich, 8091 Zurich, Switzerland; 4 Sensory-Motor Systems Lab, ETH Zurich, 8092 Zurich, Switzerland; 5 Zurich Center for Integrative Human Physiology, University of Zurich, 8057 Zurich, Switzerland; 6 Center for MR-Research, University Children’s Hospital Zurich, 8032 Zurich, Switzerland; 7 Translational Research Center, University Hospital of Psychiatry, University of Bern, 3000 Bern 60, Switzerland; 8 Institute for Biomedical Engineering, University of Zurich and ETH Zurich, 8092 Zurich, Switzerland; 9 MR Center of the Psychiatric University Hospital and the Department of Child and Adolescent Psychiatry, University of Zurich, 8032 Zurich, Switzerland; 10 Department of Psychiatry, Psychotherapy and Psychosomatics, Psychiatric Hospital, University of Zurich, 8032 Zurich, Switzerland

## Abstract

Cerebral blood flow (CBF) is related to integrated neuronal activity of the brain whereas EEG provides a more direct measurement of transient neuronal activity. Therefore, we addressed what happens in the brain during sleep, combining CBF and EEG recordings. The dynamic relationship of CBF with slow-wave activity (SWA; EEG sleep intensity marker) corroborated vigilance state specific (i.e., wake, non-rapid eye movement (NREM) sleep stages N1-N3, wake after sleep) differences of CBF e.g. in the posterior cingulate, basal ganglia, and thalamus, indicating their role in sleep-wake regulation and/or sleep processes. These newly observed dynamic correlations of CBF with SWA – namely a temporal relationship during continuous NREM sleep in individuals – additionally implicate an impact of sleep intensity on the brain’s metabolism. Furthermore, we propose that some of the aforementioned brain areas that also have been shown to be affected in disorders of consciousness might therefore contribute to the emergence of consciousness.

## Introduction

Although extensive research has been directed towards the question of why and how we sleep, until now, these issues remain largely unsolved. Amongst other hypotheses, sleep has e.g. been reported to affect responses of the immune system^1–3^ and to play a role in brain development^4^. It has also been hypothesized to conserve energy^5,6^ and has been suggested to act on the ‘downscaling’ of synaptic weights^7–9^.

While the underlying mechanisms of sleep remain unknown, sleep physiology has been well described and studied, both at baseline and in response to challenges, such as sleep restriction or sleep deprivation (i.e. the curtailment or absence of sleep). A physiological and reliable indicator of sleep pressure is slow-wave activity (SWA; power of non-rapid eye movement (NREM) sleep electroencephalogram (EEG) in the 1–4.5 Hz range), as its level reflects prior sleep-wake history. SWA is increased after sleep restriction or deprivation^10,11^ and dissipates from high levels at the beginning of sleep to lower levels in the course of sleep. SWA is also a reliable indicator of sleep intensity or sleep depth, with high levels during deep NREM sleep^12^.

Up to now, positron emission tomography (PET) and functional magnetic resonance imaging (fMRI) studies revealed mainly decreases of brain activity (assessed by regional cerebral blood flow (rCBF) or blood oxygen level-dependent (BOLD) signals) during NREM sleep, compared to waking or rapid eye movement (REM) sleep^13–18^.

Reoccurring throughout these studies, decreased brain activity was reported in subcortical areas (thalamus, brainstem, basal ganglia) and cortical regions (prefrontal cortex, anterior cingulate, precuneus). Among the brain areas that showed these decreases were neuronal populations involved in arousal (such as the basal ganglia and brainstem) and awakening, as well as structures which are most active during wakefulness (e.g. anterior cingulate, prefrontal cortex and precuneus^19,20^). The deactivation of these areas during NREM sleep may be interpreted as an involvement of sleep-promoting processes, as well as the occurrence of local cellular homeostasis. This could imply a role of NREM sleep in the recovery of brain energy, which has been hypothesized as a possible function of sleep^5,6^. Additionally, brain areas as e.g. the brainstem and the cerebellum have been shown to be involved in generation of slow waves during NREM sleep^21,22^ and showed decreases of rCBF as a function of the level of SWA^15,16^.

Slow waves (giving rise to SWA for the main part) exist as two types with distinct sites of origin (thalamic and cortical) and different underlying mechanisms of generation^23,24^. The mechanism of thalamically generated slow waves by way of intrinsic oscillations of thalamocortical neurons is quite well understood^25^. The generating mechanisms of cortically originating slow waves, however, are less clear but their existence has been demonstrated by their persistence in animals after thalamectomy^23^.

In animals, EEG slow waves have been linked to so-called up and down states intracellularly, describing periods of no action potential firing (down and off states, in single and multi unit activity, respectively) during the troughs of EEG slow waves and burst of firing (up and on states, in single and multi unit activity, respectively) during the peaks of EEG slow waves, respectively^26,27^. In epileptic patients with scalp and intracerebral EEG recordings as well as unit firing, it was recently established that the alteration pattern of burst-mode firing and silence periods during slow waves is conserved in humans as well^28^. Furthermore, this study could show that different brain areas can be in opposite states, i.e., some brain regions might be silent in an off state while at the same time other brain areas are in an on state. It is hypothesized that this burst-mode firing due to its periods of silence might be less energy demanding than tonic firing (see discussion).

Studies have proposed the frontal cortex as the initiation site of slow waves^21,22^. These results are in line with findings of PET studies that mainly associated prefrontal areas with negative correlations between rCBF and SWA (i.e. increased SWA associated with decreased rCBF) in areas such as the medial prefrontal cortex and anterior cingulate cortex^15,16^. On the other hand, a BOLD study investigating slow waves in an ‘event-related’ approach observed significant activations associated with large slow waves (>140 μV) in the brainstem, cerebellum, parahippocampal gyrus, inferior frontal gyrus, precuneus and posterior cingulate gyrus, while slow waves with smaller amplitudes (75–140 μV) were associated with frontal areas^29^. In this study, no BOLD signal decreases related to slow waves were reported.

We aimed to investigate the modulation of CBF on the one hand in relation to wakefulness and on the other hand as a function of NREM sleep depth. As prior studies suggested a connection between CBF and vigilance stage as well as a relationship between CBF and SWA, a further objective was to investigate CBF across different distinct vigilance stages (namely, pre-sleep wake, NREM sleep stages N1, N2, N3, and wake after sleep). In addition to quantifying mean CBF of different distinct, categorized wake and sleep stages and its topographical aspects, we were interested in how the temporal evolution of CBF correlated with the one of SWA during NREM sleep. Therefore, we exploited the progress that has been made in terms of imaging techniques and employed simultaneous EEG/fMRI-arterial spin labeling (ASL). ASL allows the measurement of absolute CBF, analogous to PET measurements, but with the advantage of being non-invasive and providing a much higher temporal resolution (i.e., one image approximately every 10 s compared to the PET resolution of minutes^30,31^).

Thus, we addressed two major questions. First, we wanted to confirm with fMRI-ASL recordings mean CBF differences between vigilance stages, both ‘locally’ with respect to differences in specific brain areas as well as ‘globally’ in terms of mean whole brain CBF values. Second, we were interested to establish which brain areas show a temporal relationship between CBF and NREM sleep intensity (for the first time investigating correlations between the continuous time courses of both CBF and SWA in individuals during continuous NREM sleep).

## Results

Participants included in the analyses (n = 19) were of average chronotype (no extreme morning or evening types) and showed normal levels of day-time sleepiness (Table S1).

### Mean CBF across Vigilance States

**Figure 1:**
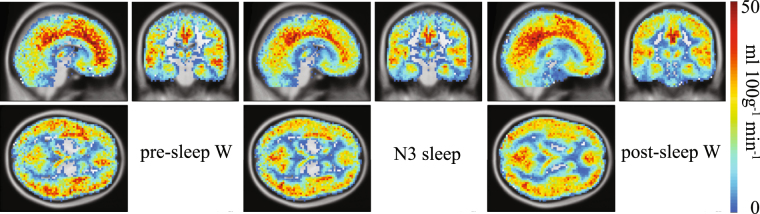
Mean cerebral blood flow (CBF) across vigilance stages. Left panel: Mean CBF across pre-sleep wake epochs (n = 16). Middle panel: Mean CBF across NREM sleep stage N3 sleep (n = 19). Right panel: Mean CBF across post-sleep wake epochs (n = 14). The absolute CBF values increase from cold to warm colors. Slices were positioned at MNI coordinates x: 6, y: −22, z: 4. Only subjects who contributed with at least 7 scans in the respective vigilance stage, i.e. ≈1 min, were included (see Table S2 for more details).

To visualize the level of topographic accuracy that can be achieved with CBF images, and to show how different the CBF is during different vigilance states even by pure visual comparison, the mean CBF is illustrated for pre-sleep wake, NREM sleep stage 3 (N3) and post-sleep wake in Fig. 1.

**Figure 2:**
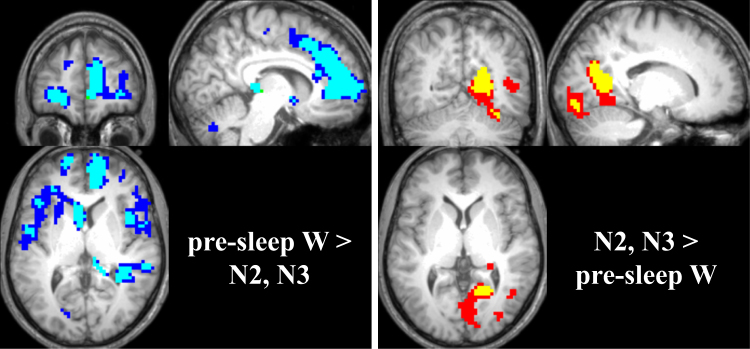
CBF during NREM sleep compared to wake. Left panel: Blue areas denote brain areas displaying lower CBF in N2 compared to pre-sleep wake; cyan areas lower CBF in N3 compared to pre-sleep wake. Slices were positioned at MNI coordinates x: 8, y: 48, z: 6. Right panel: Red areas depict areas of higher CBF during N3 compared to pre-sleep wake; yellow areas higher CBF during N2 compared to pre-sleep wake. Slices were positioned at MNI coordinates x: 16, y: −60, z: 2. All changes are displayed as p < 0.05 family-wise error (FWE) corrected with a cluster extent of 20 voxels.

**Figure 3:**
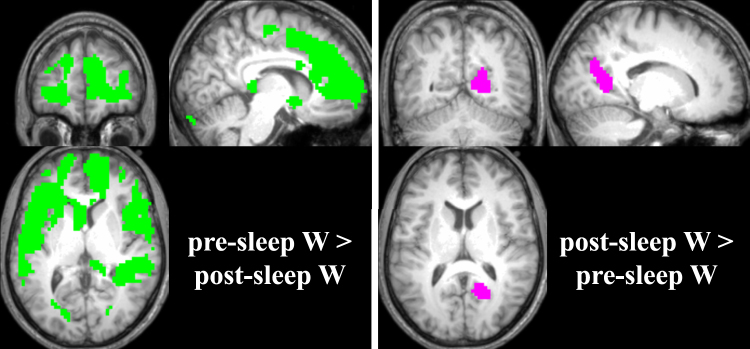
CBF differences between pre-sleep wake and post-sleep wake. Left panel: Green areas denote brain areas displaying lower CBF values during post-sleep wake compared to pre-sleep wake. Slices were positioned at MNI coordinates x: 8, y: 48, z: 6. Right panel: Pink areas depict areas of higher CBF during post-sleep wake compared to pre-sleep wake. Slices were positioned at MNI coordinates x: 16, y: −58, z: 11. All changes are displayed as p < 0.05 family-wise error (FWE) corrected with a cluster extent of 20 voxels.

Mean CBF differed in all contrasts assessed (i.e. pre-sleep W – post-sleep W, pre-sleep W - N1/N2/N3, pre-sleep W - N1, pre-sleep W - N2, pre-sleep W - N3, and the inverse of each contrast, see also Methods section; one-way within-subject ANOVA; family-wise error (FWE) corrected for p < 0.05 with a cluster extent of 20 voxels). Details are provided in Figs 2 and 3, in Tables 1 and 2, as well as in the following paragraphs.

**Table 1:** Brain regions showing significant clusters of mean CBF during NREM sleep compared to pre-sleep wake, p < 0.05 FWE corrected, cluster extent 20. Peak x, y, and z coordinates are given in MNI space with each voxels’ T value and both the hemisphere (left – L or right – R, respectively) as well as anatomical regions. Cluster denotes the total number of voxels in a given cluster, potentially combining several regions and local maxima. Anatomical information was derived using the xjView toolbox (http://www.alivelearn.net/xjview; based on the WFU_PickAtlas, http://fmri.wfubmc.edu/software/PickAtlas). In the table﻿ “aal” denotes Automated Anatomical Labeling.

Brain regions	L/R	Cluster	MNI coordinates			T value
			x	y	z	
**pre-sleep W** > **N2**						
Frontal_Sup_R (aal)	R	4432	15	59	1	13.04
Middle Frontal Gyrus	L		−21	44	−8	12.64
Frontal_Sup_Orb_L (aal)	L		−18	32	−14	12.63
SupraMarginal_L (aal)	L	141	−54	−40	34	8.90
SupraMarginal_L (aal)	L		−51	−46	25	8.27
Supramarginal Gyrus	L		−39	−49	25	7.45
Sub-Gyral (Frontal Lobe)	R	47	24	−22	43	8.19
Precentral_R (aal)	R		30	−19	55	7.48
Supp_Motor_Area_R (aal)	R		9	−19	58	5.50
Lingual_L (aal)	L	42	−18	−82	−2	7.83
Cuneus (Occipital Lobe)	L		−18	−79	7	6.56
Cerebellar Tonsil	R	36	30	−55	−41	6.97
Sub-Gyral (Parietal Lobe)	R	34	33	−52	25	6.94
Cerebelum_8_R (aal)	R	25	12	−67	−35	6.68
Precuneus	R	20	30	−49	52	6.67
Sub-Gyral (Parietal Lobe)	R		21	−46	46	5.89
**pre-sleep W** > **N3**						
Frontal_Sup_R (aal)	R	554	15	59	4	11.14
Cingulum_Mid_R (aal)	R		9	38	31	10.10
Cingulum_Ant_R (aal)	R		9	41	1	9.42
Frontal_Sup_Orb_L (aal)	L	310	−18	32	−14	10.97
Middle Frontal Gyrus	L		−21	44	−8	10.80
Frontal_Mid_Orb_L (aal)	L		−30	47	−2	9.91
Caudate_L (aal)	L	146	−6	8	7	10.44
Caudate_L (aal)	L		−12	−4	19	5.88
Middle Frontal Gyrus	R	46	33	44	−2	8.85
Frontal_Inf_Orb_R (aal)	R		27	29	−14	7.44
Frontal_Inf_Oper_R (aal)	R	93	42	11	7	8.43
Insula_R (aal)	R		45	2	−2	7.41
Extra-Nuclear (Right Cerebrum)	R		36	8	−11	5.90
Sub-Gyral (Temporal Lobe)	R	205	39	−28	−5	8.19
Sub-Gyral (Temporal Lobe)	R		36	−40	7	7.80
Temporal_Sup_R (aal)	R		57	−37	7	7.30
Frontal_Inf_Orb_R (aal)	R	37	21	17	−17	8.04
Putamen	R		18	5	−11	6.34
Putamen_R (aal)	R		21	11	1	6.13
Precentral Gyrus	R	70	45	−4	25	7.53
Precentral_R (aal)	R		48	−4	37	7.38
Rolandic_Oper_R (aal)	R		54	2	10	6.45
SupraMarginal_R (aal)	R	66	57	−28	31	7.17
SupraMarginal_R (aal)	R		48	−34	28	6.70
Postcentral_R (aal)	R		51	−25	43	6.24
Rolandic_Oper_L (aal)	L	39	−51	−1	4	7.12
Temporal_Sup_L (aal)	L		−45	−7	−5	6.18
**N2** > **pre-sleep W**						
Calcarine_R (aal)	R	167	18	−58	7	10.67
Cerebelum_6_R (aal)	R	55	27	−73	−17	9.22
Cerebelum_6_R (aal)	R		33	−58	−23	6.45
Cerebelum_6_R (aal)	R		24	−58	−17	5.41
Lingual_R (aal)	R	20	15	−85	−11	6.96
**N3** > **pre-sleep W**						
Calcarine_R (aal)	R	800	18	−58	7	11.66
Cerebelum_6_R (aal)	R		27	−73	−17	11.21
Lingual_R (aal)	R		12	−85	−11	10.62
Hippocampus_R (aal)	R	20	27	−37	1	9.35
Cerebelum_6_L (aal)	L	45	−24	−79	−20	9.32
Cingulum_Mid_L (aal)	L	21	−12	−19	40	8.54
Temporal_Mid_R (aal)	R	44	45	−61	7	7.93
Frontal_Inf_Orb_L (aal)	L	26	−48	41	−17	7.65
Middle Occipital Gyrus	R	30	33	−79	1	7.03
Occipital_Inf_R (aal)	R		33	−82	−8	6.30
Cingulate Gyrus	L	21	−12	11	34	6.65

**Table 2:** Brain regions showing significant clusters of mean CBF differences between pre-sleep and post-sleep wake, p < 0.05 FWE corrected, cluster extent 20. Peak x, y, and z coordinates are given in MNI space with each voxels’ T value and both the hemisphere (left – L or right – R, respectively) as well as anatomical regions.Cluster denotes the total number of voxels in a given cluster, potentially combining several regions and local maxima. Anatomical information was derived using the xjView toolbox (http://www.alivelearn.net/xjview; based on the WFU_PickAtlas, http://fmri.wfubmc.edu/software/PickAtlas). In the table﻿ “aal” denotes Automated Anatomical Labeling.

Brain regions	L/R	Cluster	MNI coordinates			T value
			x	y	z	
**pre-sleep W** > **post-sleep W**						
Frontal_Mid_Orb_L (aal)	L	9229	−33	47	−2	13.86
Frontal_Inf_Orb_L (aal)	L		−30	29	−14	13.68
Frontal_Sup_R (aal)	R		15	59	4	13.66
Temporal_Mid_L (aal)	L	67	−63	−46	−11	8.01
Temporal_Inf_L (aal)	L		−54	−55	−20	7.85
Calcarine_R (aal)	R	22	24	−73	4	7.30
Precuneus	L	24	−18	−49	40	6.82
Cerebelum_Crus2_R (aal)	R	33	6	−88	−26	6.64
Cerebelum_Crus2_L (aal)	L		-6	−88	−26	6.32
**post-sleep W** > **pre-sleep W**						
Precuneus	R	145	18	−58	13	10.00

### Mean CBF Maps

#### CBF during NREM Sleep Compared to Wake

Figure 2 depicts brain areas with lower CBF (left panel, cold colors) as well as higher CBF (right panel, warm colors) during NREM sleep stages N2 (cyan and yellow) or N3 (blue and red) compared to pre-sleep wake. Table 1 lists all significant clusters in detail.

Areas that showed decreases in CBF during sleep comprised e.g. the precuneus, anterior cingulate, medial frontal gyrus, parts of the basal ganglia (caudate, putamen) and the insula, as well as parts of the parahippocampal gyrus (Fig. 2, left panel; Table 1). Increases in CBF during NREM sleep stages N2 and N3 were mainly located in occipital parts of the brain such as the lingual gyrus and the cerebellum, and in a small cluster in the hippocampus (Fig. 2, right panel; Table 1).

#### CBF Differences between Pre-Sleep and Post-Sleep Wake

Figure 3 illustrates the brain areas with decreased CBF (left panel, green) as well as higher CBF (right panel, magenta) during pre-sleep wake compared to post-sleep wake. Table 2 provides all significant clusters in detail.

Significantly lower CBF during pre-sleep wake compared to post-sleep wake overlapped with the area of higher CBF during N2/N3 compared to pre-sleep wake, namely a concise part of the posterior cingulate (compare Figs 2 and 3, right panels).

Areas with higher CBF during pre-sleep wake compared to post-sleep wake were very similar to the areas that showed decreased CBF during NREM sleep N2/N3 compared to pre-sleep wake (compare Figs 2 and 3, left panels). These areas comprised the anterior cingulate, medial frontal gyrus and the caudate, as well as the bilateral superior temporal gyri, the parahippocampal gyrus and the hippocampus.

### Mean CBF Values

Mean values of whole brain and grey matter (GM) CBF (Fig. S1) differed between vigilance stages (factor ‘condition’ (pre-sleep wake, N1, N2, N3, post-sleep wake) p < 0.01, and p < 0.0001, respectively; mixed model repeated measures ANOVA). Post-hoc comparisons (Tukey-Kramer corrected for multiple comparisons) revealed that CBF was highest during pre-sleep wake, and lowest during post-sleep wake. Further, in N3, CBF was higher than during post-sleep wake both for whole brain and GM measures. GM CBF in N2 was lower than in pre-sleep wake and GM CBF in N1 was higher than in post-sleep wake (Fig. S1).

### Correlation of CBF and SWA

**Figure 4:**
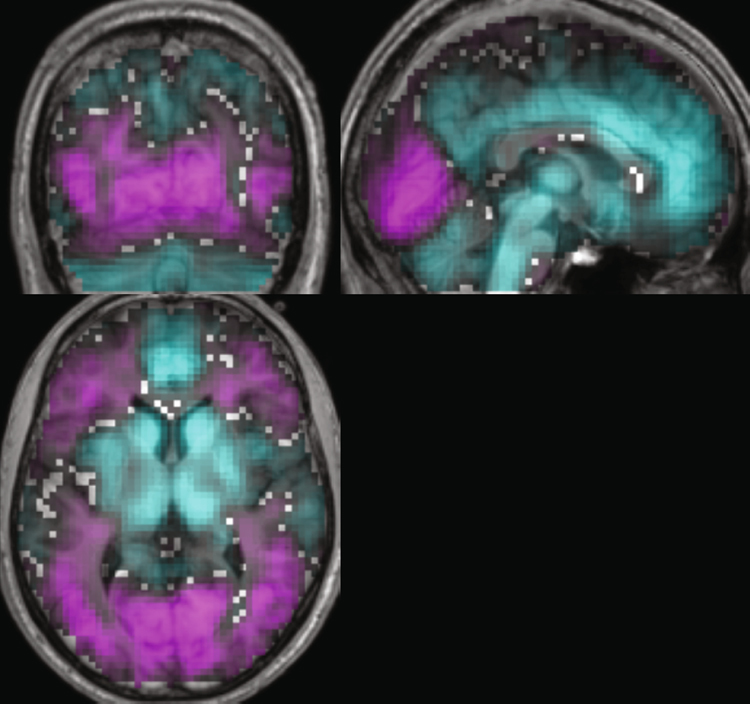
Spatial distribution of mean correlations of CBF and SWA across participants. Mean positive (pink) and negative (cyan) correlations of CBF with SWA (n = 19). Depicted are correlation coefficients ranging from 0.01 to 0.5 and −0.01 to −0.5, respectively. Slices were positioned at MNI coordinates x: 2, y: −71, z: 4.

Figure 4 displays the topographical distribution of mean correlations of CBF and SWA across participants. The correlation coefficients were derived from the partial correlations between the time course of SWA and CBF in all voxels of the brain. CBF correlated both positively (pink) and negatively (cyan) with SWA in spatially distinct brain areas. Positive correlations (increased CBF with increased SWA) were mainly located in the occipital lobe (lingual gyrus, visual cortex) whereas negative correlations (decreased CBF with increased SWA) seemed to occur more abundantly and were especially present in prefrontal areas (anterior and medial parts of the cingulate cortex), the precuneus, the basal ganglia and the thalamus. Markedly, the areas with strongest positive correlations corresponded to the areas with increased CBF during N3 compared to wake, while the most pronounced negative correlations occurred in those areas that were associated with decreased CBF during N3 compared to wake (compare Figs 2 and 3).

**Figure 5:**
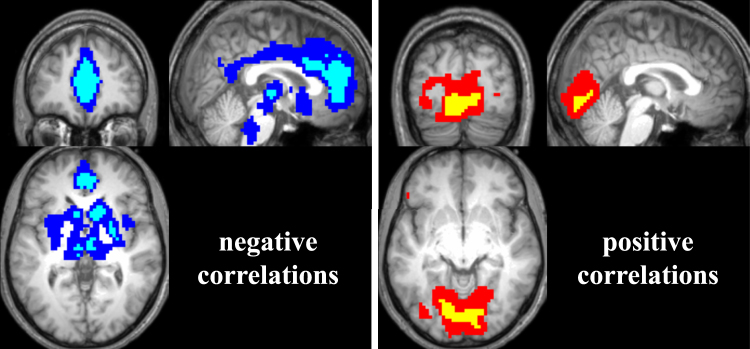
Significant correlations of CBF with SWA. Left panel: Depicted are areas where CBF was negatively correlated with SWA (blue: p < 0.01 uncorrected, cyan: p < 0.05 FWE corrected, cluster extent of 20 voxels). Slices were positioned at MNI coordinates x: 2, y: 45, z: 0. Right panel: Depicted are areas where CBF was positively correlated with SWA (red: p < 0.01 uncorrected, yellow: p < 0.05 FWE corrected, cluster extent of 20 voxels). Slices were positioned at MNI coordinates x: 2, y: −82, z: −8. 19 subjects contributed to this analysis.

CBF and SWA were significantly correlated in distinct clusters (statistical parametric maps (SPM^32^) thresholded at p < 0.05 with FWE correction for multiple comparisons and a cluster extent threshold of 20 voxels, see Table 3, Fig. 5; cyan and yellow for negative and positive correlations, respectively). Due to the lower signal to noise ratio (SNR) of the perfusion images, the SPM{t} maps were additionally thresholded at p < 0.001 uncorrected^33,34^ to display trends of the correlations, also with a cluster extent of 20 (see Table 3, Fig. 5; blue and red for negative and positive correlations, respectively).

**Table 3:** Brain regions showing significant clusters of CBF correlations with SWA, p < 0.05 FWE corrected, cluster extent 20. Peak x, y, and z coordinates are given in MNI space with each voxels’ T value and both the hemisphere (left – L or right – R, respectively) as well as anatomical region.Cluster denotes the total number of voxels in a given cluster, potentially combining several regions and local maxima. Anatomical i﻿nformation was derived using the xjView toolbox (http://www.alivelearn.net/xjview; based on the WFU_PickAtlas, http://fmri.wfubmc.edu/software/PickAtlas). In the table﻿ “aal” denotes Automated Anatomical Labeling.

Brain regions	L/R	Cluster	MNI coordinates			T value
			x	y	z	
**Negative correlations CBF SWA**						
Anterior Cingulate	R	423	6	44	7	14.96
Anterior Cingulate	L		−3	41	7	14.88
Cingulum_Ant_L	L		0	41	19	13.59
Insula_R (aal)	R	50	42	5	−8	13.60
Caudate_R (aal)	R	156	12	14	4	11.78
Sub-Gyral (Frontal Lobe)	R		21	14	−11	9.57
Medial Frontal Gyrus	R		9	17	−17	9.52
Thalamus_R (aal)	R	64	6	−10	4	9.67
Subthalamic Nucleus	L		−9	−16	−5	8.68
Midbrain	L		−6	−25	−5	8.39
Putamen	R	39	30	−10	−8	9.50
Pallidum_R			24	−1	1	9.13
Caudate_L (aal)	L	21	−6	14	1	8.55
Caudate_L (aal)			−12	11	7	8.11
**Positive correlations CBF SWA**						
Lingual Gyrus	L	305	−15	−79	−11	11.20
Lingual Gyrus	L		−3	−85	−14	9.86
Lingual_R	R		18	−70	1	9.31

Significant positive correlation clusters were located in the lingual gyrus (occipital cortex), while significant negative correlation clusters were observed in the anterior and medial frontal gyrus, in the basal ganglia (caudate and putamen), thalamus and a small cluster in the hippocampus. The positive correlations overlapped in part with the higher CBF observed during NREM sleep compared to wake (compare Fig. 2). On the other hand, negative correlations partly overlapped with regions displaying decreased CBF during NREM sleep compared to wake, such as the caudate and anterior cingulate, as well as the thalamus (compare Fig. 2). The spatial extent of the regions was comparable between correlations and stage difference images.

## Discussion

We demonstrated both local brain-area specific CBF changes and global variations of CBF during NREM sleep compared to wake. Furthermore, we could for the first time associate SWA with CBF during sleep with a very high temporal resolution (on the seconds scale) and observed both negative and positive correlations between these two measures in distinct brain areas hypothesized and known to be involved in sleep-wake regulation.

### Mean CBF across Vigilance States

We observed significant topographical differences between mean CBF images depending on vigilance stages (Figs 2 and 3). These were even visible by pure comparison (Fig. 1) and were located in areas that have already been associated with different activity during wake and sleep as e.g. the thalamus^19^.

#### CBF during NREM Sleep Compared to Wake

Contrary to the notion of sleep as a state of globally reduced brain activity, we observed higher CBF levels in occipital areas (lingual gyrus, posterior cingulate, small part of the parahippocampal gyrus and a small cluster in the hippocampus; see Fig. 2, right panel) during N2/N3 compared to pre-sleep wake. The parahippocampal gyrus has been reported to be a neuronal correlate of slow waves^29^. The authors interpreted this finding with the role of the parahippocampal gyrus as a relay station between hippocampus and neocortex and suggested an involvement in memory processing occurring during sleep. PET studies have also reported positive correlations of CBF with SWA in the primary visual and auditory cortices^16^, as well as increased CBF during SWS compared to wake^13^. These increases might be due to dream-like mentation, as suggested by Hofle *et al*.^16^ but could also be related to a preservation of functional connectivity in visual and auditory cortices as observed during anesthesia-induced loss of consciousness^35^. In their study, the authors found preserved or even higher parametric estimates (reflecting the statistical effect size) in visual and auditory networks during unconsciousness compared to wakefulness. Studies investigating disorders of consciousness as e.g. patients with unresponsive wakefulness syndrome/in vegetative state reported metabolic dysfunction in a widespread cortical network, also encompassing posterior cingulate and precuneus cortices^36,37^. Parts of this impaired network have been related to external/sensory awareness and internal/self-awareness^38^. Therefore, activity in primary cortices might thus resemble a basic form of remaining sensory processing during anesthesia or sleep that could be unique to this reversible state of unconsciousness.

Lower CBF during N3/N2 compared to wake was observed in prefrontal cortical areas (medial frontal gyrus, anterior cingulate) and in the basal ganglia (caudate, putamen; see Fig. 2, left panel) as well as in part of the thalamus. These areas have previously been reported to show decreased CBF in sleep compared to wake based on PET studies^13,17,20^.

The prefrontal cortex has been proposed as the initiation site of cortically generated slow waves^21,22^. The decrease of CBF in this area could be due to the burst-mode firing of cortical neurons generating up and down states that give rise to slow waves at the EEG level^27^. This burst-mode is characterized by longer periods of ‘silence’ (off periods/down states) followed by short activity bursts (on periods/up states), in contrast to the tonic firing during waking. Therefore, during NREM sleep, when CBF is averaged over seconds or minutes the net result of off and on periods might be reduced overall neuronal activity, manifested in a decrease in CBF compared to waking.

The basal ganglia (in particular the striatum) have been proposed to play a role in sleep-wake regulation^39^, as they constitute part of the ascending reticular arousal system (ARAS) located in the brainstem^40^. The ARAS integrates signals from e.g. cortical areas as well as from the thalamus and amygdala. Stroke or atrophy affecting the caudate have been associated with distinctive changes in sleep patterns (hypersomnia and reduced SWS, respectively^41^), and cats with removed caudate showed transient hyposomnia^42^. Based on its anatomical connectivity, less caudate activation would lead to inhibition of the globus pallidus interna (therefore resulting in higher activation of it), which in turn would cause more inhibition of the thalamic nuclei. This reduced activation of the thalamic nuclei (which is manifested in our data in a reduction of the pulvinar activation) would then decrease excitation of the frontal cortex.

Together, these data suggest a role of the basal ganglia, the prefrontal cortex, the posterior cingulate and the thalamus in sleep-wake regulation, as well as a prefrontal cortex role in slow wave generation. Our data further corroborate such a notion as we observed NREM sleep associated decreases of CBF in the aforementioned brain areas compared to wakefulness. As some of these areas (e.g. the thalamus, prefrontal cortex) are also among those showing an impaired metabolism in patients with unresponsive wakefulness syndrome/ in the vegetative state^36,37^ but not in patients in emergence from minimally conscious state^38^, implications might extend towards neuronal underpinnings of consciousness and its restoration (see related interpretations by He and Raichle^43^).

#### CBF Differences between Pre-Sleep Wake and Post-Sleep Wake

The brain regions with lower CBF during pre-sleep wake compared to post-sleep wake (see Fig. 3, right panel) included the area that displayed increased CBF during NREM sleep (compare Fig. 2, right panel), namely the posterior cingulate. Similarly, higher CBF during pre-sleep wake compared to post-sleep wake was mainly observed in regions associated with decreased CBF in NREM sleep (compare Figs 2 and 3, left panels), i.e., in the anterior cingulate, medial frontal gyrus, the basal ganglia and the parahippocampal gyrus. Pre-sleep wake was additionally associated with higher CBF in the bilateral superior temporal gyri and part of the supplementary motor area.

These brain-region specific effects mainly resemble the patterns observed in the sleep-wake contrast. Together with the fact that we did not observe differences in mean grey matter CBF between N3 and post-sleep wakefulness (in contrast to^13^; see section “Mean GM CBF Values” and the SI Discussion), this could imply a remaining influence of NREM sleep on post-sleep wakefulness. As we did not restrict post-sleep wake to a period after prolonged continuous consciousness of a certain duration (as Braun and colleagues did), our results could thus be attributed to sleep inertia. This would be in line with a study by Balkin *et al*.^44^ that reported a distinct time sequence of ‘awakening’ for specific brain regions. In detail, these authors observed a fast ‘recovery’ of CBF values during wake after sleep to values observed during pre-sleep wake in the brainstem and thalamus but showed that reestablishment of pre-sleep wake CBF took longer in anterior cortex regions. In particular, the caudate region associated with lower CBF during post-sleep wakefulness compared to pre-sleep wake is in favor of such an interpretation: Although this caudate region showed also lower CBF during N3 compared to pre-sleep wake, the spatial extent was larger compared to the contrast of pre-sleep and post-sleep wake. This would be in line with the report of Balkin *et al*.^44^ that CBF in these areas returns faster to pre-sleep wake levels than e.g. prefrontal cortical areas.

### Mean GM CBF Values

To assess more global changes of CBF in the different vigilance stages, we compared mean GM and whole brain CBF values between them. Mean GM CBF values differed significantly between vigilance stages (Fig. S1). This is in line with previous reports^13^. However, the changes observed in mean GM CBF values with respect to vigilance stages were not completely in accordance to those observed by Braun *et al*.^13^. However, the levels of CBF during pre-sleep wakefulness in this paper were comparable to those found in a recent ASL study^45^. Several differences between the two studies might have contributed to the divergent findings. These issues are discussed in detail in the SI Discussion.

### Correlation of CBF and SWA

We observed significant clusters of correlations between CBF and SWA in distinct brain areas (Fig. 5). When uncorrected for multiple comparisons, these clusters became larger and revealed additional related brain areas (e.g., anterior and medial portions of the cingulate, occipital cortex etc.; Fig. 5). For the first time, we could demonstrate that a temporal relationship between CBF and SWA during continuous NREM sleep exists in individuals, thereby establishing a dynamic link between metabolic (CBF) and electrophysiological (sleep intensity) measures. This extends the findings of PET studies that correlated CBF and SWA values measured at distinct time points during different sleep stages pooled across subjects^15,16^.

These areas with the positive correlations (higher levels of CBF associated with higher SWA) overlapped with areas shown to have higher CBF values during N2/N3 sleep than during pre-sleep W. As discussed above, this might indicate e.g. either dream-like mentation processes during sleep or constitute a form of remaining basic level of stimulus processing during sleep, possibly due to the underlying unique state of reversible unconsciousness.

Brain areas associated with negative correlations (lower levels of CBF associated with higher SWA) on the other hand overlapped in anterior cingulate and basal ganglia regions with areas displaying lower CBF values during N2 sleep (and additionally thalamus regions during N3 sleep) compared to pre-sleep W. Some of the overlaps of these two analyses might be inherent to the fact, that SWA is highest during NREM sleep, and tends to increase from N2 to N3 sleep. However, the nature of these analyses (distinct, categorized, visually scored 20-s stages grouped together with potentially very different underlying levels of SWA (see CBF, hypnogram and SWA in SI-Fig. 2), averaged per subject and contrasted with pre-sleep wake versus the correlation of each subjects’ continuous temporal evolution of CBF and SWA throughout ongoing NREM sleep; SI-Fig. 2) is quite different. Further, NREM sleep is not exclusively characterized by slow waves and SWA in stages N2/N3 but also consists of more complex frequency compositions (see e.g. the spectrogram in SI-Fig. 2) including features such as K-complexes and sleep spindles.

Therefore, although the scoring of NREM sleep and SWA are not completely orthogonal, we are confident that the correlation analysis furthers our understanding of brain regions involved in sleep-wake regulation and slow wave generation. This is also illustrated in SI-Fig. S2 that shows while SWA and NREM sleep stages share some properties, the correlation analysis provides additional information about dynamical changes occurring within sleep that cannot be assessed by the static contrasting of average CBF in a specific sleep stage with pre-sleep wake.

The negative correlations between CBF and SWA in a small thalamic cluster are in line with the results presented by Hofle *et al*.^16^. As mentioned, some of these areas display metabolic dysfunctions in patients with unresponsive wakefulness syndrome/ in the vegetative state^36–38^ but not in patients emerging from minimally conscious state^38^. Therefore, these areas might not only contribute to sleep-wake regulation but could further be related to the subsiding and restoration of consciousness.

Additionally, a small cluster in the hippocampus was associated with significant negative correlations of CBF and SWA. The hippocampus is part of the memory consolidation system and it has been proposed that sleep might amongst others serve a function in the scope of memory processes^32,46,47^. A study employing intracranial EEG recordings in epilepsy patients linked increases in hippocampal EEG power in the high delta range (2.1–4 Hz) after training and performance improvement in a hippocampus-dependent spatial navigation task the next day^48^. Both slow waves^28^ and spindles^49^ have been shown to occur locally in the hippocampus during sleep. As mentioned above, slow wave generation is due to a burst-mode of firing alternating with periods of silence that is hypothesized to be less energy demanding than tonic firing. As this process seems also to take place in the hippocampus, this could be the reason for the observed negative correlation between hippocampal CBF and SWA.

## Conclusions

In their entirety, our data corroborate the involvement of thalamic, basal ganglia and prefrontal cortex areas in sleep-wake regulation and strengthen the notion of their association with different states of consciousness. Further, we were able to demonstrate that specific brain areas (namely the posterior cingulate) showed an increase of CBF during NREM sleep compared to wake. By combining comparisons of mean CBF across vigilance stages and a temporal correlation analysis of continuous CBF with SWA (a marker of sleep intensity), we corroborated that these brain areas do not only change CBF in sleep with respect to a baseline (usually pre-sleep wake) but that there is a close relationship between specific temporal neuronal patterns (as assessed by SWA) and the corresponding CBF. Additionally, we showed that CBF during immediate post-sleep wake mainly resembles NREM sleep-like features, with the exception of the caudate region which seemed to ‘recover’ to pre-sleep wake values faster the other brain areas, in line with its presumed role in awakening and arousal.

## Material and Methods

### Participants

The study was approved by the ethical committee of the Canton of Zurich and was conducted according to the Helsinki declaration. Participants gave their written informed consent to participate in the study and were remunerated for their participation.

Twenty-four healthy male participants were selected for the study after a thorough screening process excluding excessive daytime sleepiness (defined as values >10, assessed by the Epworth Sleepiness Scale (ESS^50^; range: 0–24, 0: lowest possible level of daytime sleepiness, above 10: excessive daytime sleepiness), and neurological or psychiatric disorders (based on subjective reports). Participants also had to be compatible to the magnetic resonance (MR) safety regulations as well as in the normal body mass index (BMI) range (World Health Organization (WHO) normal range: 18.5–24.99 kg/m2; http://apps.who.int/bmi/index.jsp?introPage=intro_3.html). Participants further had to report regular sleep-wake patterns involving approximately 8 h of sleep per night (Table S1). All participants were moderate caffeine (≤3 cups/day) and alcohol (≤7 alcoholic drinks/week) consumers, and were not on medications.

Participants underwent a screening night in the sleep laboratory and only those who reached at least 80 % sleep efficiency and could sleep on their back without sleep fragmentation were included in the study. Demographics and behavioral measures of the participants that were included in the subsequent analyses (n = 19, exclusion criteria provided below) are provided in Table S1.

### Study Protocol

Three days prior to the first experimental night, participants had to adhere to regularly scheduled bedtimes and time in bed of 8 hours, compliance to this was determined based on both individual sleep diaries and wrist actigraphy recordings (Actiwatch; Cambridge Neurotechnology, Cambridge, UK).

Participants spent the night prior to the scanner recordings in the sleep laboratory. They went to bed at 23:30 and high density (hd) EEG recordings were performed. They had a sleep opportunity of 4 h, and were awakened at 03:30 in the morning. They had to stay awake until the next evening, when the scanning session took place. From 03:30 to 07:00, they had to stay in the laboratory and were under constant supervision. Compliance throughout the day was monitored with wrist actigraphy.

At the scanner site, participants underwent a resting state EEG recording outside of scanner, two resting state recordings (simultaneous EEG/fMRI-BOLD- and EEG/fMRI-ASL measurements, respectively) and performed a modified Sternberg working memory task^51^. The working memory data will not be assessed in this publication. Afterwards, at ≈23:30, participants were instructed to sleep and to signal if they either were not able to go back to sleep after awakening or if they wanted to abort the experiment completely. If participants only slept for a short duration (≈2 h), after a short break (≈0.5 h) they were asked whether they would be ready to sleep in the scanner again. After completion of the sleep scanning sessions, the EEG cap was removed and anatomical as well as diffusion weighted tensor images were recorded.

### fMRI Data Acquisition and Preprocessing

The simultaneous EEG/fMRI-ASL sleep recordings were performed with a 3T Philips Achieva whole-body system (Philips Medical Systems, Best, The Netherlands) with a 32-element receive-only head coil (Philips SENSE head coil 32-elements). ASL images were obtained with 2D EPI readouts with a pseudo-continuous labeling scheme with the following parameters: 72 volumes, a 80 × 79 matrix, a slice thickness of 7 mm with no gap, 3 × 3 × 7 mm^3^ voxels, 20 slices, repetition time/ echo time /label time/post label delay (TR/TE/τ/PLD) of 4400/20/1650/1525 ms, a flip angle of 90°, a field of view of 240 mm, a labeling offset of 2 cm from the bottom imaging slice (corresponding roughly to 9 cm offset from the anterior commissure-posterior commissure (AC-PC) line) and background suppression. Duration of the recordings varied depending on participants’ sleep duration (see above) but was set maximally to 1600 scan volumes (i.e. ≈4 h).

To quantify CBF^52^, an equilibrium magnetization volume (M0) was acquired right before the fMRI-ASL recordings, with the same parameters as for the fMRI-ASL sequence described above, except that a longer TR of 10000 ms and no labeling was applied. ASL slices were oriented along the AC-PC line such that the whole cortex was recorded. Therefore, the cerebellum was not fully covered with the selected slices.

The fMRI-ASL data were first realigned in SPM8 http://www.fil.ion.ucl.ac.uk/spm/software/spm8/. ASL data were quantified to CBF images (MATLAB version R2012b, The MathWorks Inc., Natick, Massachusetts). The approach is based on the simple subtraction method as specified in Aguirre *et al*.^53^. This method has been described to reliably minimize BOLD contamination in resting state recordings^54^. CBF images were then co-registered, normalized to Montreal Neurological Institute (MNI) space (and resliced to 3 × 3 × 3 mm^3^ isomorphic voxels), masked (with participant specific brain masks) and smoothed (6 mm full-width at half maximum (FWHM) Gaussian Kernel) with SPM8.

Individual grey matter (GM) masks were obtained by segmentation of the anatomical images in SPM8. For the GM images, participants’ GM masks were normalized to MNI space. Participants’ pre-processed mean CBF images of each vigilance stage were masked with the individual GM mask.

### EEG Data Acquisition and Preprocessing

The concurrent EEG was acquired with an MR compatible amplifier and electrodes (BrainAmp, BrainCap and BrainAmp ExG MR devices and electrodes; Brain Products GmbH, Gilching, Germany). The data were sampled at a 5 kHz and synchronized with the scanner clock. We recorded 66 channels (60 EEG channels, 2 electrooculogram (EOG) channels, 1 electromyogram (EMG) channel, 3 electrocardiogram (ECG) channels). The analog filter settings were as follows: low-pass at 250 Hz, high-pass at 0.0159 Hz (time constant 10 s).

For the further EEG analyses, scanner gradient artifacts were removed by a fast Fourier transform (FFT) approach (adapted from a method used in electron crystallography, collaboration with R. Dürr, in house MATLAB script) and down-sampled to 500 Hz. Data were inspected for movement artifacts and corrected with a standard cardioballistogram (CB) artifact template correction implemented in BrainVision Analyzer 2 (Brain Products GmbH, Gilching, Germany; Allen *et al*., 1998).

Furthermore, the EEG data were filtered (band-pass filter: high-pass cutoff at 0.1 Hz, low-pass cutoff at 49 Hz, additional notch filter at 50 Hz) and an independent component analysis (ICA) derived artifact filter was applied. For most participants (n = 15), the ICA filter was constructed from those independent components (ICs) that were manually selected as artifacts of an ICA performed on combined data sets of resting state ASL recordings (≈10 min, free of movement artifacts) and the ‘clean’ EEG from the resting state outside of the scanner (≈8 min, free of movement artifacts). These resting state data had been obtained prior to the sleep data during rested wake in the same subjects at the same scanner. For the remaining four participants, the ICA filters were constructed from the manual selection of ICs derived from an ICA of ≈4 min of resting state EEG outside of the scanner and ≈4 min of the sleep recordings right at the beginning (free of movement artifacts). For the pre-sleep wake EEG data, the aforementioned filter created from the resting state ASL recordings and the ‘clean’ EEG was used.

Based on the cleaned EEG recordings, sleep was visually scored (derivations C3A2, F3A2, O1A2; A2 approximated by closest electrode in the EEG cap) in 20-s epochs according to standard guidelines (wake, NREM sleep stages 1 (N1), N2, N3^55,56^).

### Inclusion/Exclusion Criteria for Participant Data and Analyses Details

To be able to assess CBF across the whole span of vigilance stages, only participants who reached NREM sleep stage N3 according to standard scoring criteria^55^ were included in the analyses (n = 19). We indicate also if fewer subjects contributed to an analysis due to insufficient number of scans of a particular vigilance stage.

#### Mean CBF across Vigilance Stages

To assess the mean CBF values per vigilance state (wake or sleep), we averaged across all scans (minimum number of 7 scans, i.e. ≈1 min) of the respective artifact free sleep scans. Movements related to scans were identified based on the frame-wise displacement (FD) data. Scans with an FD values greater than 0.5 were excluded^57^ (details see below). The resulting mean maps were then averaged per participant, to reveal a single mean CBF value per participant per stage. Wake before sleep (pre-sleep W), NREM stages N1, N2, N3, and wake after sleep (post-sleep W) were compared. Wake before sleep was calculated from those scans of the resting state recording prior to the sleep recording.

#### Correlation of CBF and SWA

When two scanning sessions had been recorded in a participant, only one of them was included in the analyses (typically, the session where NREM sleep stage N3 had been reached and if this was the case in both sessions, the one with less artifacts was selected). Sleep duration differed between participants (170.3 ± 59.9 min in total; mean ± standard deviation (SD), range from 63.7 to 234.3 min).We selected NREM sleep recordings, that comprised at least NREM sleep stages N2 and N3 and as little movement artifacts as possible, and were at least 1 hour long (see Table S1).

### Statistical Analyses

#### Mean CBF across Vigilance Stages

Mean global CBF values (grey matter, GM) of each participant and vigilance stage (pre-sleep W, N1, N2, N3, post-sleep W) were analyzed with a mixed model repeated measures ANOVA implemented in SAS (SAS Institute Inc., Cary, North Carolina). Post-hoc comparisons were based on the Tukey-Kramer test.

Mean CBF images of each participant and vigilance stage entered a second level SPM factorial design with a one-way within-subject ANOVA. Participants contributed only with those conditions, i.e. stages, for which they had enough artifact free scans (≥7). In detail, 14 participants contributed to the condition ‘pre-sleep W’, 18 to ‘N1’, 19 to ‘N2’ as well as ‘N3’ and 14 to ‘post-sleep W’ (Table S2). The following contrasts were assessed: pre-sleep W – post-sleep W, pre-sleep W - N1/N2/N3, pre-sleep W - N1, pre-sleep W - N2, pre-sleep W - N3, and the inverse of each contrast. We only report contrasts pre-sleep W – post-sleep W, pre-sleep W – N2, pre-sleep W N3 and the respective inversions.

#### Correlation of CBF and SWA

EEG power density spectra were calculated from the sleep EEG recordings using an FFT routine (4-s epochs, Hanning window), averaging 2 4-s epochs centered at the mid-point of the ASL label and control scans (i.e., 4.4 s after start of label, 8.8-s intervals). The resulting SWA (power in the 1–4.5 Hz range of EEG derivation C3A2; A2 approximated by closest electrode in the EEG cap) time course was smoothed with a moving average across 21 8.8-s intervals (Fig. S2).

Likewise, the CBF time course of every voxel in the brain was smoothed with a moving average across 21 scans (8.8-s intervals). Based on an approach of Power *et al*.^57^, we excluded all scans that showed an FD values greater than 0.5. The FD value was derived by a combination of all six movement parameters obtained from the realignment of the functional images.

Artifact–corrected CBF time courses of all voxels in the brain and the corresponding SWA time course were entered in a partial correlation analysis, with nuisance regressors of no interest. These nuisance regressors were the FD values determined by the motion parameters of the realignment step of the functional images, as well as 16 white matter and 16 cerebrospinal fluid regressors derived with the CONN functional connectivity toolbox^58^. These parameters have been shown to give good estimates of physiological noise contributions due to pulsation and breathing artifacts. These regressors were also smoothed with a moving average filter of 21 intervals.

The resulting partial correlation coefficient images of each participant were Fisher’s z-transformed. These z-transformed images were entered in a second level SPM factorial design with a one-sample T-test (two-tailed). Positive and negative contrasts, i.e. positive and negative correlations of SWA and CBF were assessed.

Figure 4 illustrates the mean correlation (n = 19) between CBF and SWA. Averaging was performed on participants’ z-transformed correlation images and then backtransformed for display.

### Data availability

The ethical approval granted to the authors by the ethical committee of the Canton of Zurich (Switzerland) does not allow the publication of the raw data online. If readers would like to reanalyze the data set (for different purposes), additional ethical approval (on a individual user and purpose basis) will be required. The authors would be happy to support additional ethical approval applications from researchers for access to this data set.

## Electronic supplementary material

Supplementary material
