# Supplementary material for: In human non-REM sleep, more slow-wave activity leads to less blood flow in the prefrontal cortex
Source: Sci Rep. 2017 Nov 3;7:14993. doi: 10.1038/s41598-017-12890-7 (PMC5670199; doi:10.1038/s41598-017-12890-7)
Supplement: Supplementary file 1 — Supplementary material [file 41598_2017_12890_MOESM1_ESM.pdf]

Supplementary Material

In human non-REM sleep, more slow-wave activity leads to less blood flow in the prefrontal cortex

Authors: Laura Tüshaus, Ximena Omlin, Ruth O'Gorman Tuura, Andrea Federspiel, Roger Luechinger, Philipp Staempfli, Thomas Koenig, Peter Achermann

SI Discussion

Mean GM CBF Values

Similar in both our and the study of Braun et al. [1] was that CBF decreased in wake after sleep (post-sleep W) compared to wake before sleep (pre-sleep W). However, Braun and colleagues found a marked decrease of mean CBF in slow wave sleep (SWS; NREM sleep stages 3 and 4) compared to pre-sleep wake, and further showed post-sleep wake mean CBF to be significantly higher than CBF in SWS [1]. These findings are corroborated by our results of pre-sleep/post-sleep wake and N2 which show the same direction of decrease/increase as Braun and colleagues [1]. However in contrast, we observed higher CBF during N3 compared to post-sleep W and no difference between CBF of pre-sleep W and N3. Also, the absolute ranges of mean CBF values are different. These discrepancies are puzzling and need further discussion.

Several differences between the two studies may have contributed to the divergent findings. First of all, in the study of Braun and colleagues [1], subjects were considerably more sleep deprived (24–54 h) while the subjects investigated in our study were only subjected to a sleep restriction of 4 h the night prior to scanning, resulting in prolonged wakefulness at the time of the sleep recording of ≈ 20 h. As increased sleep pressure has been shown to increase brain activity in subjects who are able to remain alert after sleep deprivation [2], this might have resulted in the higher mean CBF value observed by Braun and colleagues during wake and led to significant difference between wake and SWS. In favor of this notion is that in both studies, mean CBF values during SWS and N3, respectively, range around 40 $ml \cdot 100\ g^{-1} \cdot min^{-1}$. Recent studies have shown mean GM CBF values in rested wake to fluctuate in the same range as we have observed (see Fig. 2 [3]).

Yet, if the absolute CBF values would be comparable across the studies, this would still not explain why CBF at post-sleep wake differed. What might have contributed to the discrepancy might be the number of subjects contributing to each stage and the time point of assessment. Braun and colleagues [1] obtained post-sleep wakefulness CBF values in scans collected after 15min or more of continuous wakefulness. In our study, we defined post-sleep wake as any epoch scored as 'wake' after consolidated sleep. Therefore, effects of sleep inertia could have influenced the CBF at post-sleep W obtained by us, while Braun and colleagues might have captured already the process of awakening.

Further, Braun et al. [1] measured in general more subjects (number of subject reported in Braun et al. [1] and reported in this study: pre-sleep wake 32/16, SWS: 22/18, post-sleep wake: 22/14 subjects).

Last, CBF values might not be absolutely comparable between PET and ASL studies. Although quantification of absolute CBF has been shown to be reliable with ASL techniques [4, 5] the differences in temporal resolution might contribute to substantially disparate findings, as the time frame of the scans attributed to EEG activity, i.e., stage, was markedly different.

Functionally, the decrease of mean CBF during sleep has been attributed to a less energy demanding state of the brain, due to burst-mode firing of pyramidal neurons involved in the generation of slow waves [6]. This could imply that one function of sleep is to incorporate conservation and restoration of brain energy.

As Braun and colleagues [1] did not investigate CBF in N1 and N2, comparisons are not possible. However, the decrease of mean CBF in N2 compared to wake but no difference in CBF between W and N3 could reflect changes in brain connectivity reported by Spoormaker et al. [7]. They observed a sharp decrease in thalamo-cortical connectivity in N1 that was partially reestablished in deeper sleep stages (so connectivity between the thalamus and the cortex was reduced at sleep onset but strengthened again in N3), paralleled by increases of cortico-cortical connectivity in N1 that were subsequently suspended in SWS.

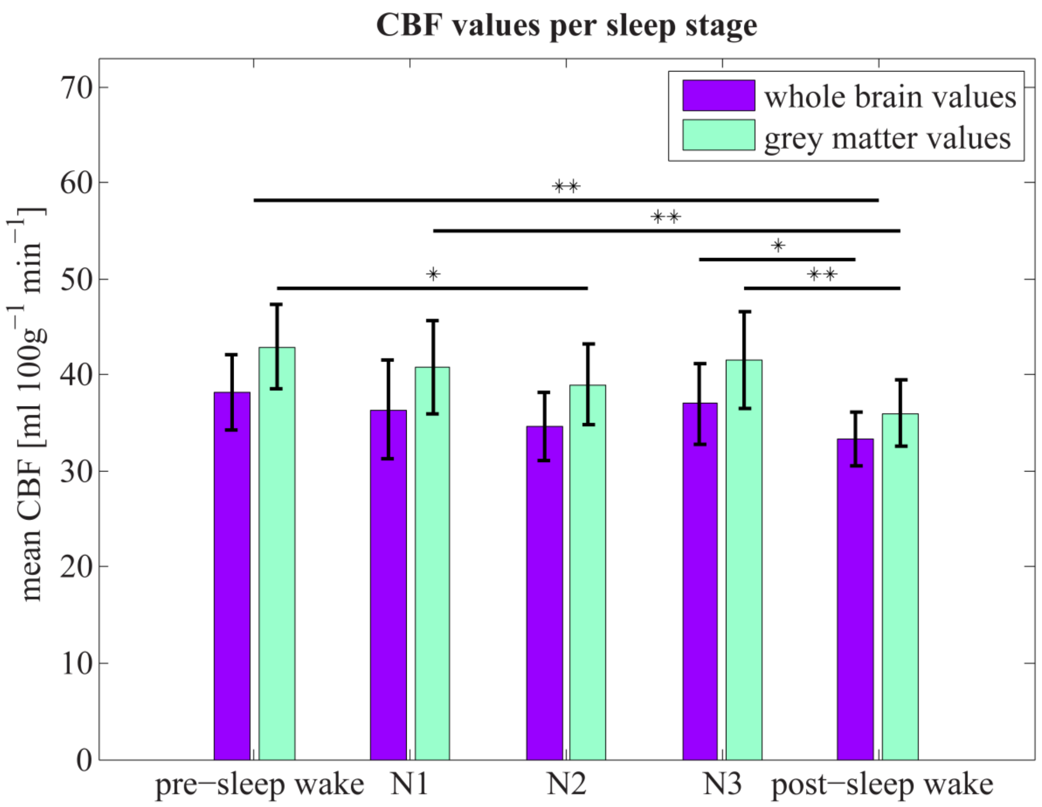

Fig. S1: Bar plot illustrating mean CBF values across different vigilance stages. Bars depict absolute CBF values ([ml·100 g^{-1}· min^{-1}], mean ± standard deviation). Stars indicate significant differences between vigilance stages. ∗: $p < 0.05$, ∗∗: $p < 0.01$, Tukey-Kramer test, corrected for multiple comparisons. Violet bars indicate whole brain CBF values, turquoise bars represent grey matter CBF values.

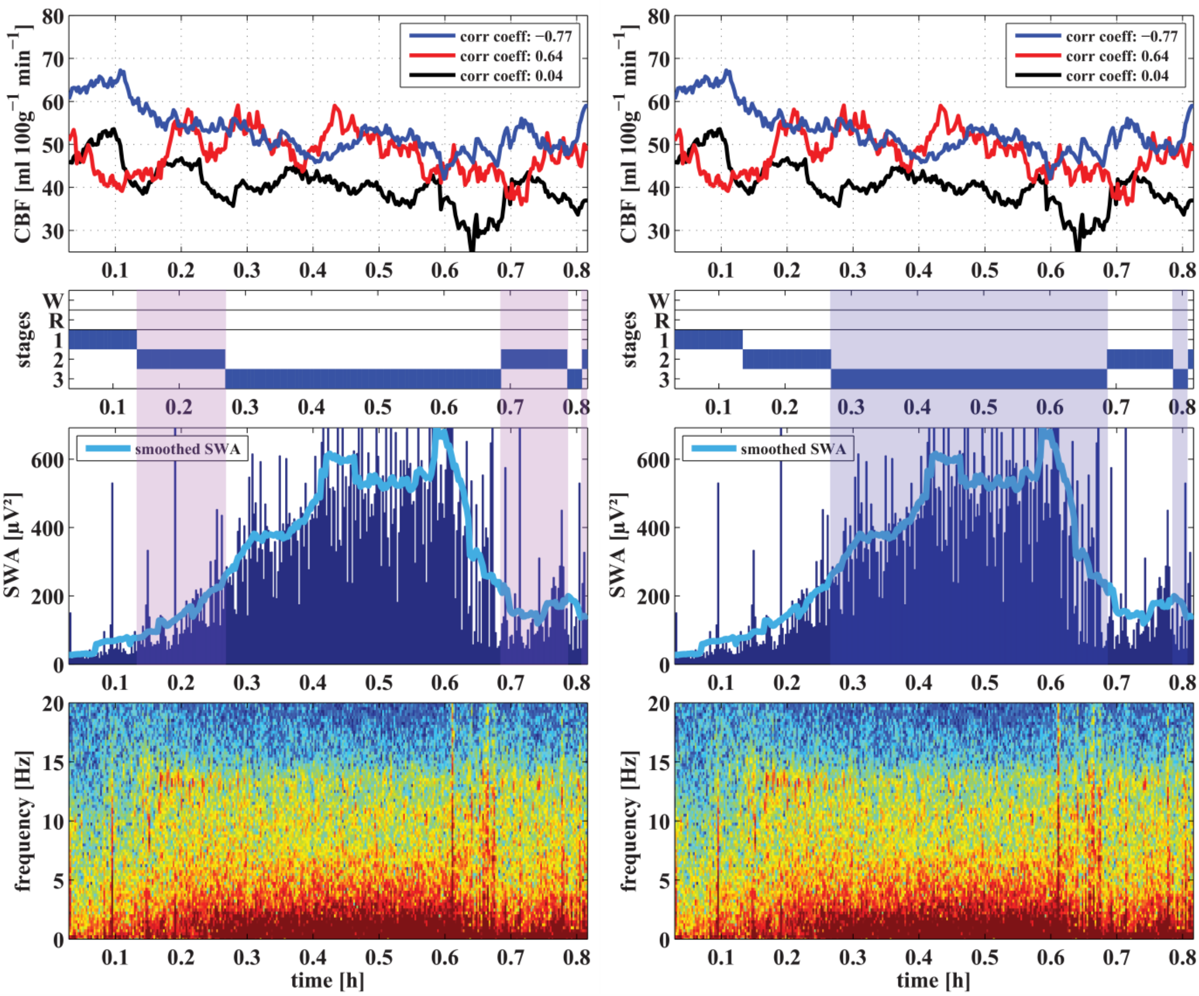

Fig. S2: Schematic illustrating the partial correlation of CBF and SWA. Top panel: Exemplary CBF time courses of three voxels (smoothed with moving average filter, n = 21). The voxel time course depicted in blue is anti-correlated with SWA, the voxel time course in red is positively correlated with SWA and the black voxel time course does not show a meaningful correlation. Stated are the partial correlation coefficients (see Material and Methods for detailed information about these correlations). Middle panels: Hypnogram (W: wake; R: REM sleep; 1 – 3: NREM sleep stages N1 – N3) and SWA (i.e., power in the range of 1–4.5Hz). In light blue, the smoothed SWA time course (moving average filter, n = 21) is shown. Bottom panel: Spectrogram (color-coded for power density spectra) across the analyzed NREM sleep episode. Warm colors denote high power density in the respective frequencies, cold colors low power density. Data were obtained at 8.8-s intervals.
Left: Shaded areas indicate stage N2; right: stage N3. Please note, that SWA varies considerably even in a given sleep stage. Especially towards the end of the cycle, SWA levels during N3 are much lower and comparable to those in N2 than in the middle of the cycle. Further, up to approximately 0.2 hours, SWA during N2 and N1 (very beginning of the cycle, not shaded) is at comparable levels. Therefore, this illustrates that the correlation analysis adds complementary information about dynamical changes within sleep to the static contrasting of average CBF in a specific sleep stage with pre-sleep wake.

Variables	Mean ± SD (if applicable)
**No. of subjects**	19
**Age [years]**	22.5 (2.3)
**BMI [kg/m^2]**	22.8 (2.2)
**ESS**	4.6 (2.3)
**Mid sleep free days corrected (MCTQ) [hh:mm]**	3:45 (0:46)
**Average habitual sleep duration (MCTQ) [hh:mm]**	7:34 (0:31)
**Range of habitual sleep duration (MCTQ) [hh:mm]**	6:17 – 8:28
**Duration of NREM sleep episodes to determine correlations [min]**	170.3 (59.9)

Tab. S1: Demographic and behavioral characteristics. The mean is displayed plus/minus the standard deviation (SD), if applicable. BMI: body mass index, ESS: Epworth Sleepiness Scale, MCTQ: Munich Chronotype Questionnaire.

stage	No. of Scans (± SD)	No. of Subjects
**pre-sleep W**	38.1 (18.6)	14
**N1**	83.7 (85.8)	18
**N2**	584.3 (264.6)	19
**N3**	299.2 (178.5)	19
**post-sleep W**	64.5 (65.6)	14

Tab. S2: Number of scans and number of participants per vigilance stage. Scans had a duration of 8.8s, i.e., were obtained at 8.8-s intervals. SD: standard deviation, pre-sleep W: wake before sleep, N1 – N3: NREM sleep stages, post-sleep W: wake after sleep.

References

1. Braun, A. R. et al. Regional cerebral blood flow throughout the sleep--wake cycle An H2(15)O PET study. Brain 120, 1173-1197 (1997).
2. Poudel, G. R., Innes, C. R. H. & Jones, R. D. Cerebral Perfusion Differences Between Drowsy and Nondrowsy Individuals After Acute Sleep Restriction. Sleep 35, 1085-1096 (2012).
3. Ghisleni, C. et al. Effects of Steroid Hormones on Sex Differences in Cerebral Perfusion. PloS One (2015).
4. Aslan, S. et al. Estimation of Labeling Efficiency in Pseudocontinuous Arterial Spin Labeling. Magn Reson Med 63, 765-771 (2010).
5. Borogovac, A. & Asllani, I. Arterial Spin Labeling (ASL) fMRI: Advantages, Theoretical Constrains and Experimental Challenges in Neurosciences. International Journal of Biomedical Imaging 2012, 818456 (2012).
6. Vyazovskiy, V. V. et al. Cortical Firing and Sleep Homeostasis. Neuron 63, 865-878 (2009).
7. Spoormaker, V. I. et al. Development of a Large-Scale Functional Brain Network during Human Non-Rapid Eye Movement Sleep. The Journal of Neuroscience 30, 11379-11387 (2010).
